# SIRT1 mediates the inhibitory effect of Dapagliflozin on EndMT by inhibiting the acetylation of endothelium Notch1

**DOI:** 10.1186/s12933-023-02040-x

**Published:** 2023-11-28

**Authors:** Weijie Wang, Yilan Li, Yanxiu Zhang, Tao Ye, Kui Wang, Shuijie Li, Yao Zhang

**Affiliations:** 1 Department of Cardiology, The Second Affiliated Hospital of Harbin Medical University, 246 Xuefu Road, Harbin, 150086 China; 2 Key Laboratory of Myocardial Ischemia, Ministry of Education, Harbin Medical University, Harbin, China; 3 Department of Biopharmaceutical Sciences, College of Pharmacy, Harbin Medical University, Harbin, 150081 China; 4 State Key Laboratory of Frigid Zone Cardiovascular Diseases (SKLFZCD), Harbin, China; 5 Department of Organic Chemistry, College of Pharmacy, Harbin Medical University, Harbin, China

**Keywords:** Heart failure, Endothelial–mesenchymal transition, SGLT-2 inhibitors, SIRT1, Deacetylation

## Abstract

**Background:**

Endothelial–mesenchymal transition (EndMT) plays a crucial role in promoting myocardial fibrosis and exacerbating cardiac dysfunction. Dapagliflozin (DAPA) is a sodium–glucose-linked transporter 2 (SGLT-2) inhibitor that has been shown to improve cardiac function in non-diabetic patients with heart failure (HF). However, the precise mechanisms by which DAPA exerts its beneficial effects are yet to be fully elucidated.

**Methods:**

Isoproterenol (ISO) was used to generate a HF model in mice. For in vitro experiments, we used TGF-β1-stimulated human umbilical vein endothelial cells (HUVECs) and mouse aortic endothelial cells (MAECs).

**Results:**

Both our in vivo and in vitro results showed that EndMT occurred with decreased SIRT1 (NAD^+^-dependent deacetylase) protein expression, which could be reversed by DAPA therapy. We found that the protective effect of DAPA was significantly impaired upon SIRT1 inhibition. Mechanistically, we observed that SIRT1 phosphorylation, a required modification for its ubiquitination and degradation, was reduced by DAPA treatment, which induces the nucleus translocation of SIRT1 and promotes its binding to the active intracellular domain of Notch1 (NICD). This interaction led to the deacetylation and degradation of NICD, and the subsequent inactivation of the Notch1 signaling pathway which contributes to ameliorating EndMT.

**Conclusions:**

Our study revealed that DAPA can attenuate EndMT induced by ISO in non-diabetic HF mice. This beneficial effect is achieved through SIRT1-mediated deacetylation and degradation of NICD. Our findings provide greater insight into the underlying mechanisms of the therapeutic effects of DAPA in non-diabetic HF.

**Graphical Abstract:**

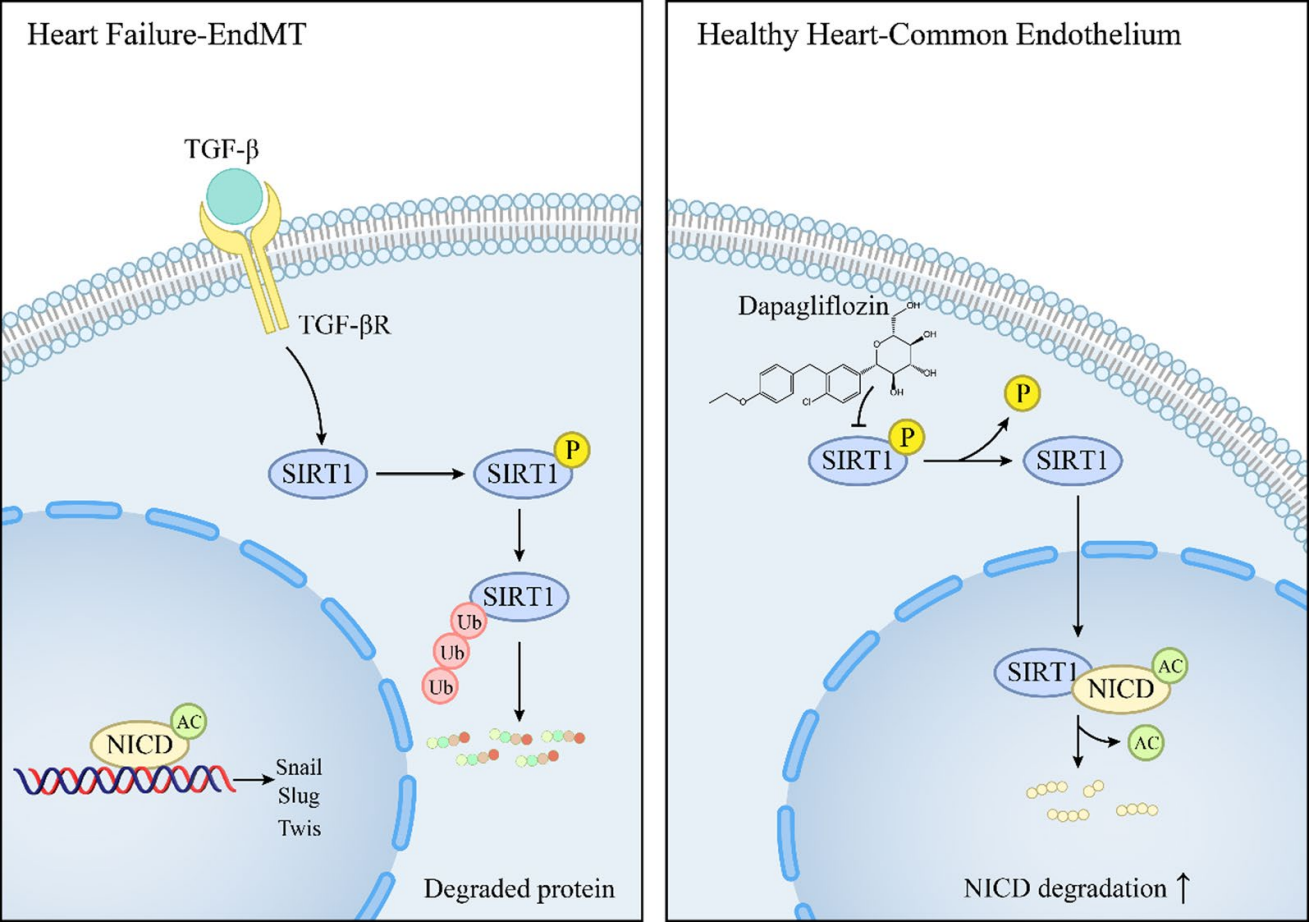

**Supplementary Information:**

The online version contains supplementary material available at 10.1186/s12933-023-02040-x.

## Introduction

Myocardial fibrosis is a critical pathological alteration in heart failure (HF) development. While previous studies have primarily focused on the role of fibroblast activation in myocardial fibrosis [1], researchers have often overlooked the crucial contribution of endothelial cells to the progression of HF. It is worth noting that approximately 30% of cardiac fibroblasts are derived from endothelial cells, where EndMT occurs [2]. EndMT refers to the transformation of endothelial cells into mesenchymal cells, accompanied by changes in the expression of various transcription factors and cytokines, including increased cell migration and invasion, cell translocation, and extracellular matrix production [3]. Stimulated by various pathological factors, endothelial cells differentiate into fibroblasts that secrete excessive extracellular matrix into the heart and perivascular tissues through the EndMT process, leading to excessive collagen deposition and promoting the progression of myocardial fibrosis [4]. Several studies have shown that EndMT inhibition reduces cardiac fibrosis [5–7]. Therefore, targeting EndMT represents a crucial approach for effectively suppressing myocardial fibrosis.

Dapagliflozin (DAPA), a sodium–glucose linked transporter 2 (SGLT-2) inhibitor, was originally developed to regulate blood glucose levels in patients with type 2 diabetes. The DAPA-HF trial, the first to investigate the impact of SGLT-2 inhibitors on HF outcomes in patients with or without type 2 diabetes (HFrEF), demonstrated that DAPA significantly reduced the risk of worsening HF (hospitalization or an urgent visit resulting in intravenous therapy for HF) or cardiovascular death in HFrEF patients when added to standard HF treatment [8]. In addition, several preclinical studies have substantiated the favorable impact of DAPA on endothelial cells, suggesting its potential role in alleviating endothelial dysfunction and microvascular injury by inhibiting oxidative stress and inflammatory response [9, 10]. Despite these promising findings, the effect of DAPA on EndMT in non-diabetic HF patients and the underlying mechanisms of myocardial fibrosis remain poorly understood. A deeper understanding of these mechanisms will provide valuable insights into the potential clinical applications of DAPA in treating non-diabetic HF patients.

SIRT1 is a class III histone deacetylase that relies on nicotinamide adenine dinucleotide as a cofactor [11–13]. SIRT1 deacetylates both histone and non-histone proteins and plays critical roles in various cellular processes, including apoptosis, autophagy, energy metabolism, cell differentiation, anti-aging, and DNA damage repair. In cardiovascular diseases, SIRT1 has been shown to protect against cardiomyocyte apoptosis, maintain endothelial function, and attenuate myocardial fibrosis through a variety of pathways [14, 15]. It has been found that SIRT1 inhibits cardiomyocyte apoptosis, protects endothelial function, and alleviates myocardial fibrosis through a variety of pathways [16]. Interestingly, DAPA has been reported to activate SIRT1 expression and inhibit angiotensin-induced SIRT1 downregulation in cardiomyocytes [17]. However, whether and how DAPA activates SIRT1 in endothelial cells remains unknown. Previous studies have demonstrated that SIRT1 activation inhibits EndMT and improves cardiac and renal fibrosis [18, 19]. In light of these findings, we sought to investigate whether DAPA confers its protective effects on EndMT and fibrosis in non-diabetic HF mice through the activation of SIRT1 and to further elucidate the underlying molecular mechanisms.

## Materials and methods

### Animal model establishment and groupings

Adult male C57BL/6 mice aged 5 weeks were purchased from the Laboratory Animal Center of the Second Affiliated Hospital of Harbin Medical University (Harbin, China). Before the experiment, the animals were kept in sterile filter-top cages and subjected to a 12-h light/dark cycle at 22 ± 3 °C and 45 ± 10% humidity for 7 days.

The mice were randomly divided into four groups: vehicle-treated (vehicle); isoproterenol (ISO) treatment; ISO plus DAPA gavage (ISO + DAPA); and ISO plus DAPA and EX527 combined gavage (ISO + DAPA + EX527). There were 6 mice in each group. Details of the group dose and administration route are provided in Additional file 1.

### Echocardiography

Echocardiography was performed using a GE/Vingmed Ultrasound Vivid7 (GE Healthcare, Little Chalfont, UK). Left ventricular fractional shortening (LVFS) and left ventricular ejection fraction (LVEF) were calculated.

### Histopathological staining

Hematoxylin and eosin staining (Solarbio, China), picric acid-Sirius red staining (Phygene, China), and Masson trichrome staining (Solarbio, China) were used for histological and fibrosis analysis.

### Immunohistochemistry

Immunohistochemistry (IHC) was performed as described [20]. The following antibodies were used in IHC: a-SMA and CD31.

### Single-cell dataset download and quality control

From GEO downloads the single-cell data sets for humans (GSE121893 and GSE109816, containing 10 heart tissue samples). “Create Seurat Object” function of the Seurat R package (v4.1.0) was used to construct the Seurat object data set single-cell data, and the number of cells, number of genes, and percentage of mitochondria were quality-controlled using IQR (interquartile range). Detailed analysis methods for single-cell sequencing data are provided in Additional file 1.

### Processing microarray data

Datasets GSE82294 and GSE36074 were obtained from the Integrated Gene Expression Database (www.ncbi.nlm.nih.gov/geo).GSE82294 included 178 samples (divided into Ctrl, ISO, and ISO + ATE treatments). GSE36074 included 19 samples (5 for sham surgery, 7 for HF after TAC surgery, and 7 for no HF after TAC surgery). We analyzed the HF group and Ctrl or sham group.

### Cell culture and treatments

Human umbilical vein endothelial cells (HUVECs, zqxzbio, ZQ0446) and mouse aortic endothelial cells (MAECs, BNCC, BNCC359881) were cultured in DMEM (Gibco, USA).

The cells were treated with TGF-β1 (10 ng/mL) for 48 h to establish the EndMT model. For DAPA treatment, the cells were pretreated with DAPA (10 μM) for 2 h and incubated with TGF-β1 for 48 h with or without EX527 (10 μM). For SIRT1 knockdown in vitro, a small interfering RNA (siRNA, Gemma gene, China) was synthesized, and a random sequence was synthesized as a negative control. Lipofectamine™3000 (Invitrogen, USA) was used for 48 h to transfect cells according to the manufacturer’s instructions.

### Protein extraction and western blotting

To obtain total protein extracts, cultured cells or frozen heart tissue were lysed in Radio Immunoprecipitation Assay (Beyotime Biotechnology, China) at 4 °C. The protein concentration of the supernatant was determined using the BCA method (Beyotime Biotechnology, China). After the sample was completed, SDS-PAGE gel electrophoresis was performed, and a protein-free rapid sealing solution was used after transmembrane. The primary antibody was incubated overnight, and the membrane was washed before the secondary antibody was incubated. The membranes were washed and then developed with an ECL luminometer.

### Immunofluorescent staining

For immunofluorescent staining of HUVECs and MAECs, the cells were washed with PBS and fixed with paraformaldehyde at room temperature. Cells were incubated in goat serum (Boster, China) and then incubated with primary antibodies overnight at 4 °C. Cells were washed three times and incubated with secondary antibodies for 1 h at room temperature. Nuclei were stained with DAPI (Beyotime Biotechnology, China) for 5 min at room temperature.

### Molecular docking

The 3D structure of the SIRT1 protein was obtained from the RCSB database (PDB ID: 4IG9). AVOCADO 1.2.0 was used to minimize the energy of the DAPA 3D structure under the MMFF94 force field. In this study, AutoDock Vina 1.1.2 software was used for molecular docking.

### Protein–protein docking

CLUB PRO was used to perform a docking with the SIRT1 protein as the receptor and the Notch protein as the ligand under default parameters, and the highest-ranked docking conformation output was considered to be the bound conformation. PyMol Academic open-source edition was used to visualize the docking results. PDBePISA was used for online analysis of the docking results to study the binding energy of the SIRT1 and Notch proteins.

### Transwell cell migration assay

Transwell experiment was carried out in each group after the corresponding treatment. Transwell was performed as described [21].

### EdU cell proliferation assay

Cell proliferation was determined using an EdU kit (Ribobio, China). Proliferating cells exhibited bright red fluorescence under a laser confocal microscope (Leica, Japan).

### Analysis of SIRT1 function and KEGG signaling pathway enrichment

In this study, a PPI network for SIRT1 was constructed using the STRING database, which provides key integrations of PPIs, including known and predicted interactions. Interaction pairs with high confidence (combined score > 0.4) were selected to construct the PPI network. The KEGG signaling pathways involved in SIRT1 regulation were enriched through the DAVID website.

### Co-immunoprecipitation (Co-IP) protein immunoprecipitation

Cells in each group were carefully washed twice with precooled PBS, and NP-40 cell lysis buffer (Beyotime Biotechnology, China) was added. According to the protein concentration, the antibody was added to the supernatant as instructed and mixed overnight at 4 °C on a rotating oscillator. The supernatant of each protein was added to the tube containing only protein A/G agarose bead suspension (Bimake, USA), and the sample was slowly shaken on a rotating oscillator at 4 °C for more than 4 h. The magnetic beads were adsorbed using a magnetic stand, and the precipitate was retained and washed three times with precooled NP-40.

### Nucleus/cytoplasm protein separation assay

Use the nucleus protein and cell plasma protein extraction kit (Beyotime Biotechnology, China) to separate the nucleus and cytoplasm protein.

### DAPA-SIRT1 binding assays

To assess the binding of DAPA to SIRT1, we utilized biotin labeled small molecule compounds and pull-down assay. For the pull-down assays, we used PierceTM Biotinylated Protein Interaction Pull-Down (Thermo Fisher). In strict accordance with the specifications. The eluted products were further verified by Western blotting experiments.

### Cellular thermal shift assay (CETSA)

Based on the principle that target proteins usually become stable when bound to drug molecules, we used CETSA to verify the binding of DAPA and SIRT1. The DMSO or DAPA-treated cell lysates were divided into ten equal parts and boiled at temperatures ranging from 37 to 64 °C. After freezing and thawing liquid nitrogen twice, the supernatant was collected for follow-up experiments.

### Statistical analysis

All data analysis was performed with Prism 8.0 (GraphPad). One-way ANOVA followed by Tukey’s post hoc test for multiple comparisons was utilized for statistical analyses. Each experiment was repeated at least 3 times, and data were shown as means ± standard deviation (SD). The results were considered significant when the *P* value < 0.05, and *P* values < 0.01 were considered highly significant.

## Results

### DAPA ameliorates ISO-induced myocardial perivascular fibrosis and cardiac dysfunction by impairing EndMT

**Fig. 1:**
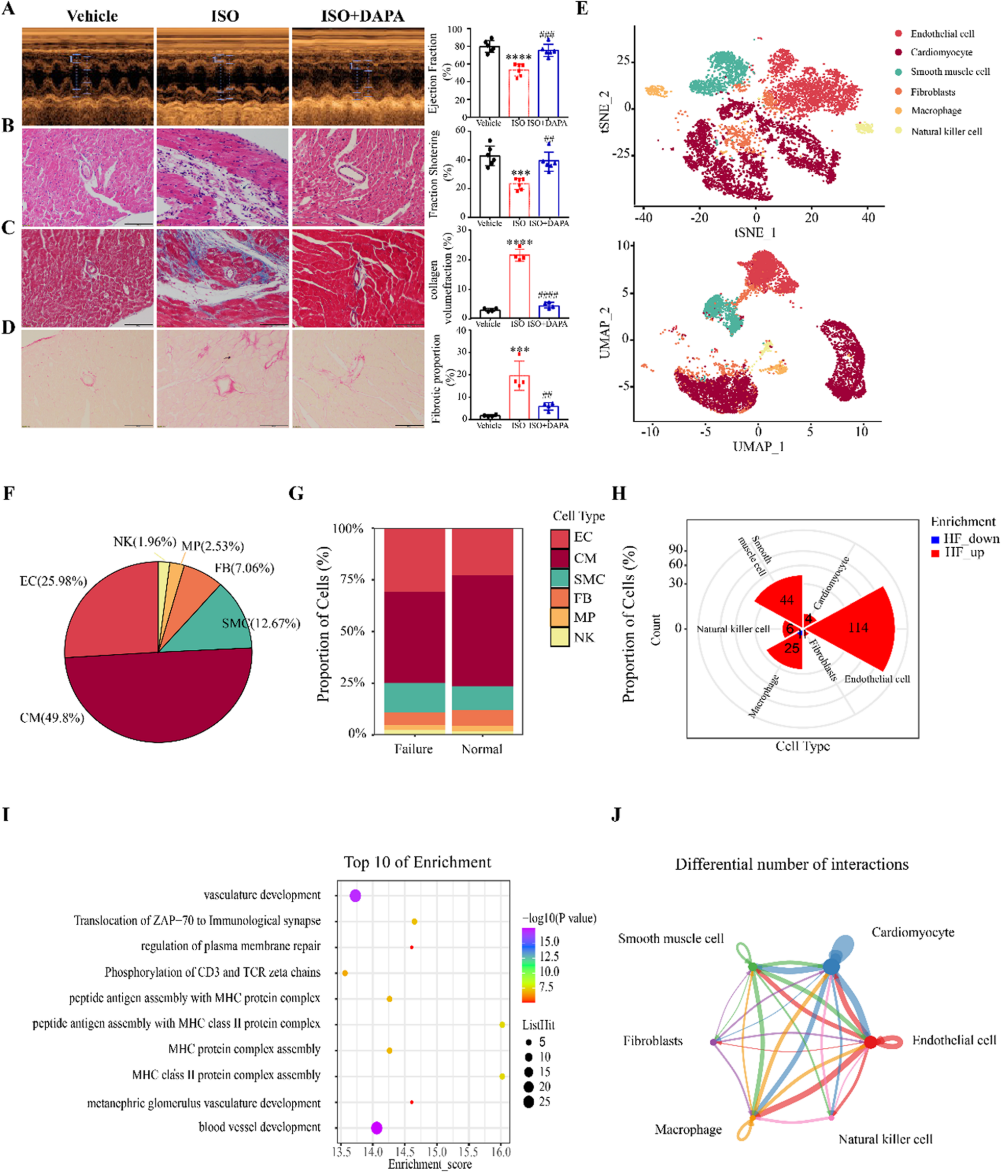
DAPA ameliorates ISO-induced myocardial perivascular fibrosis and cardiac dysfunction by targeting endothelial cells. **A** Images of transthoracic echocardiograms in M-mode (n = 6). **B**, H&E staining of the lengthwise section of the heart. Scale bar = 100 μm. **C** Representative images showing Masson staining of perivascular fibrosis in heart sections. Scale bar = 100 μm. **D** Representative images of perivascular fibrosis stained with PSR, and red represents collagen fiber deposition. Scale bars = 100 μm. **E** tSNE plots and UMAP plots. Each point depicts a single cell, colored according to cluster designation. **F** The proportion of the corresponding cell types. **G** Cell proportion of 2 sample sources in each cell type. **H** Rose diagrams showing the differentially expressed genes (DEGs) numbers after HF in each cell type. **I** Top 10 of GO terms and pathways enriched in DEGs of HF and Normal. **J** Circle plot showing the total intercellular signaling network in the HF group. Blue indicates weakening, while red indicates strengthening after HF. ^*^p < 0.05, ^**^p < 0.01, ^***^p < 0.001 and ^****^p < 0.0001 vs. Vehicle group; ^#^p < 0.05, ^##^p < 0.01, ^###^p < 0.001 and ^####^p < 0.0001 vs. the ISO group

We administered ISO by subcutaneous injection to construct a mouse myocardial fibrosis model to investigate the effects of DAPA on cardiac function and myocardial fibrosis in mice. The left ventricular ejection fraction (LVEF) and the left ventricular fractional shortening (LVFS) are the most commonly used indexes of left ventricle contractile function. Therefore, these two metrics were chosen to evaluate the cardiac function of the mice in each group. Echocardiography showed that ISO-treated mice had a lower EF and FS, and impaired left ventricular systolic function than control mice (Fig. 1A). Upon DAPA treatment, the systolic function of the heart was rescued to normal levels. Morphologically, we observed severe myocardial tissue damage in the ISO group (Fig. 1B). Masson trichromatic staining and picric acid-Sirius red (PSR) staining showed that ISO significantly increased collagen deposition in mouse myocardial and perivascular tissues. Meanwhile, DAPA treatment significantly alleviated these pathological changes (Fig. 1B–D).

We analyzed previously published datasets of single-cell sequencing in HF patients. Each cell type was annotated according to the expression level of typical cell-type-specific markers (Fig. 1E). Endothelial cells from HF patients made up 25.98% of all cells (Fig. 1F). Endothelial cells showed significant differences in cell types and gene expression after HF (Fig. 1G–I). In addition, there was enhanced communication between endothelial cells and different cells in the late stages of HF (Fig. 1J and Additional file 1: Figure S1), suggesting that endothelial cells may play a crucial role in HF. Based on the above findings, we presumed that the pathological changes of endothelial cells may play a central role in chronic HF. Therefore, our study focused on the function of DAPA in regulating myocardial fibrosis by altering the pathological state of endothelial cells.

**Fig. 2:**
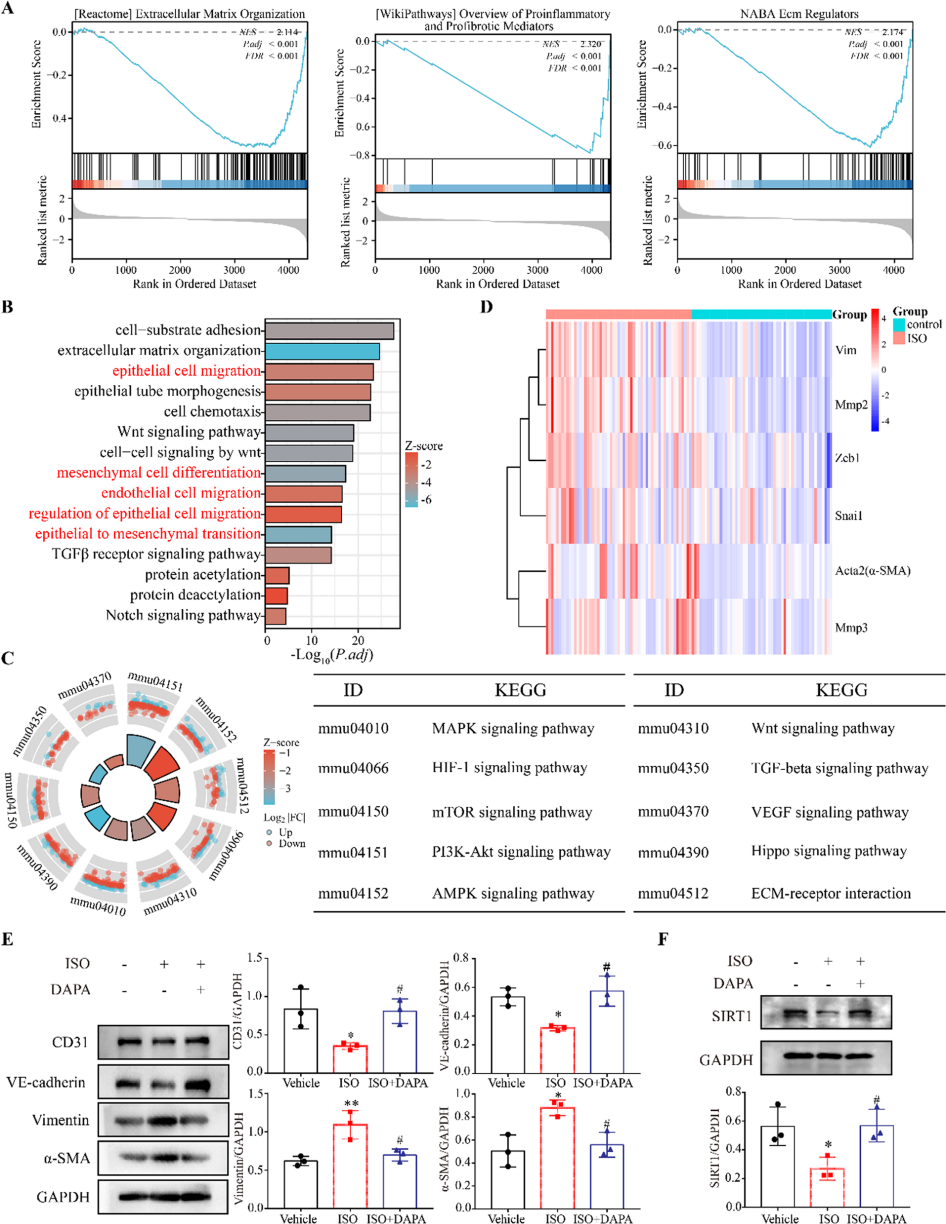
DAPA ameliorates ISO-induced myocardial perivascular fibrosis and cardiac dysfunction by impairing EndMT. **A** Results for the GSEA enrichment of DEGs. **B** Enrichment analysis of biological processes involved in the regulation of EMT or EndMT-related pathways. **C** Enrichment analysis of KEGG signaling pathways involved in regulating EMT or EndMT-related pathways. **D** Enrichment of EMT or EndMT marker genes. **E** The Western blotting analysis of EndMT-related proteins and quantitative analysis (n = 3). **F** The Western blotting analysis of SIRT1 and quantitative analysis (n = 3). *p < 0.05, **p < 0.01, ***p < 0.001 and ****p < 0.0001 vs. Vehicle group; #p < 0.05, ##p < 0.01, ###p < 0.001 and ####p < 0.0001 vs. the ISO group

We analyzed a data set related to ISO-induced HF in mice (GSE82294). GESA analysis showed that DEGs were significantly associated with extracellular, immune, and extracellular matrix regulation with “Extracellular Matrix Organization”, “Overview of Proinflammatory and Profibrotic Mediators”, and “Ecm Regulators” (Fig. 2A). Biological processes and KEGG were enriched in “epithelial-stromal transformation”, “endothelial cell migration”, “epithelial cell migration”, and EndMT-related signaling pathways (Fig. 2B and C). EndMT-related transcription factors and marker genes were significantly up-regulated in ISO (Fig. 2D). Similar results were shown for the TAC mouse model dataset (Additional file 1: Figure S2). We investigated the protein expression of the EndMT marker in mouse hearts treated with ISO and DAPA. Our findings demonstrated a significant reduction in endothelial cell markers (CD31 and VE-cadherin) and an increase in mesenchymal cell markers (α-SMA and Vimentin) upon ISO treatment, which were partially restored following DAPA administration (Fig. 2E). Previous studies have indicated that SGLT-2 inhibitors exert their protective effects on HF partly through SIRT1 activation [22, 23]. Interestingly, our results revealed a significant restoration of SIRT1 expression levels by DAPA treatment (Fig. 2F).

### DAPA inhibits TGF-β1-induced EndMT in HUVECs and MAECs

**Fig. 3:**
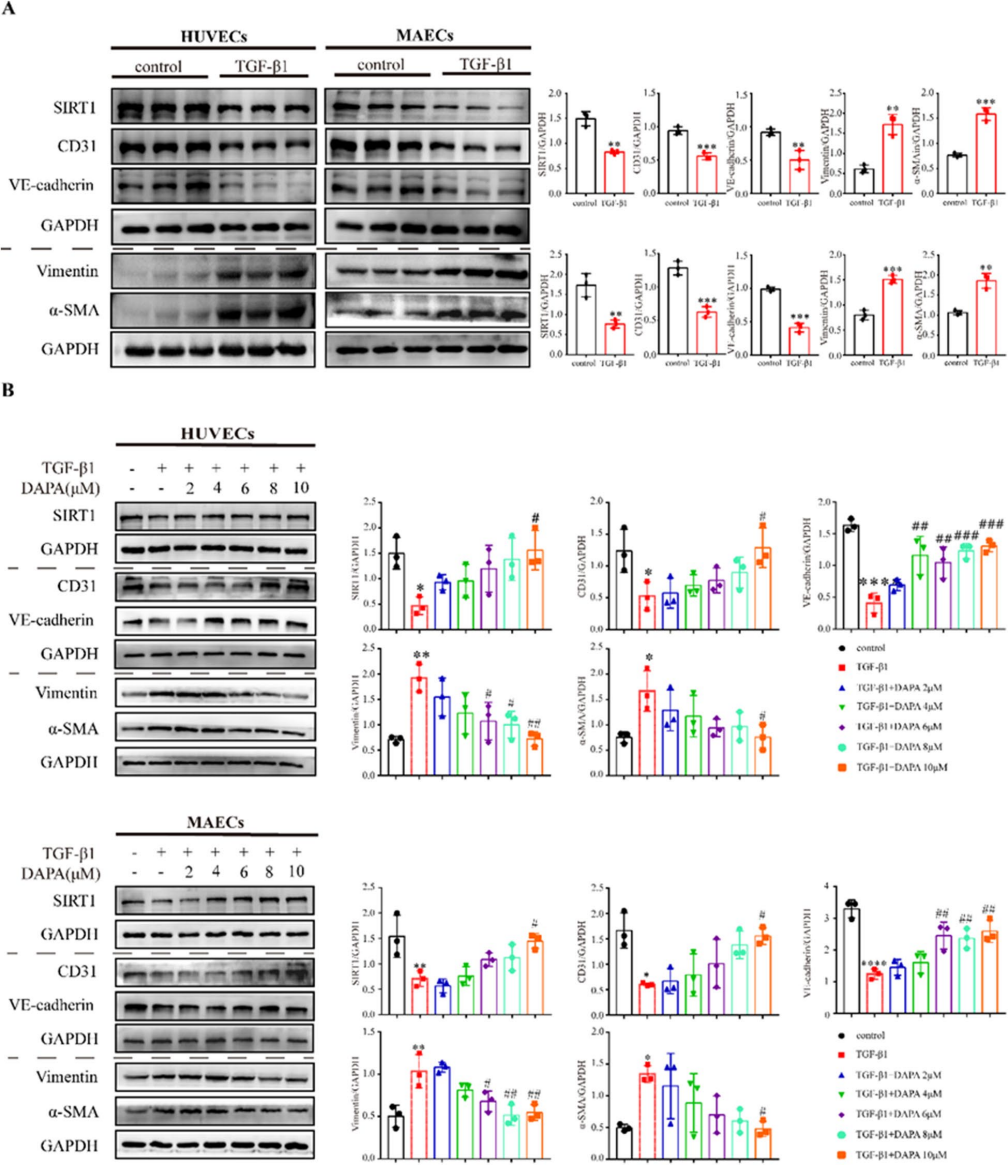
DAPA inhibits TGF-β1-induced EndMT. **A** Western blotting analysis of HUVECs and MAECs treated with TGF-β1 for 48h and the quantitative results. **B** A representative Western blotting and quantitative analysis of EndMT markers in HUVECs and MAECs treated with DAPA for 48h with or without TGF-β1. *p < 0.05, **p < 0.01, ***p < 0.001 and ****p < 0.0001 vs. control group; #p < 0.05, ##p < 0.01, and ###p < 0.001 vs. TGF-β1 group

To further elucidate the underlying mechanism of DAPA regulation on EndMT, we employed TGF-β1 to induce an EndMT model in HUVECs and MAECs. We observed that TGF-β1 promoted EndMT (Fig. 3A) and combined treatment with DAPA significantly decreased the expression of Vimentin and α-SMA and increased the expression of CD31 and VE-cadherin in a dose-dependent manner as compared to the TGF-β1 group (Fig. 3B). Furthermore, we discovered that SIRT1 expression was suppressed upon TGF-β1 treatment but restored by pretreatment with DAPA (Fig. 3B).

### SIRT1 is required for DAPA-mediated protection of endothelial cells in EndMT

**Fig. 4:**
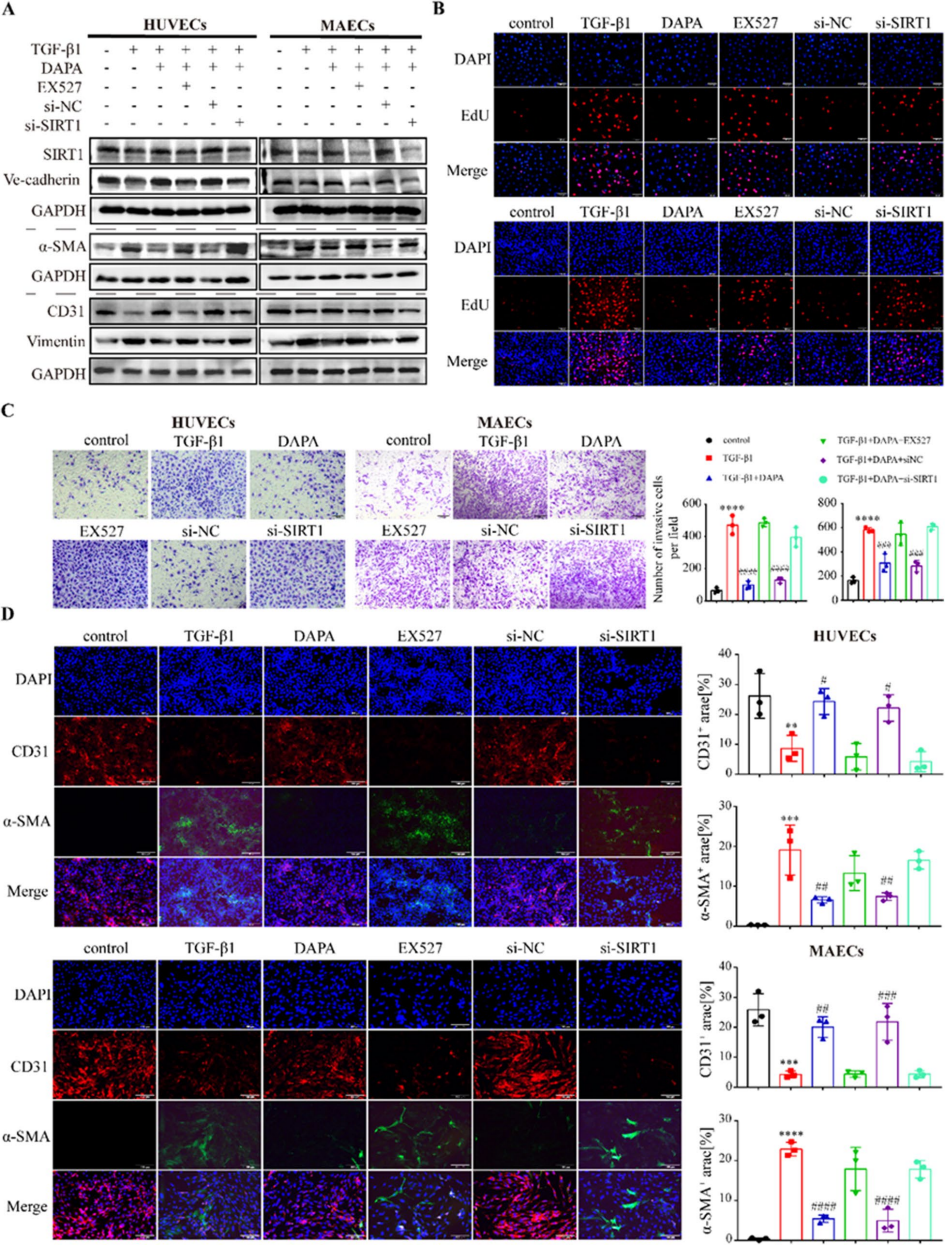
SIRT1 is involved in DAPA-mediated protection of endothelial cells in EndMT. **A** Western blotting analysis of EndMT markers and SIRT1 in HUVECs and MAECs treated as indicated (The statistical results are presented in Additional file 1: Figure S3A). **B** Representative images of the EdU proliferation assay of HUVECs and MAECs treated as indicated. Red fluorescence indicates proliferating cells (the statistical results are presented in Additional file 1: Figure S3B). Scale bars = 100 μm. **C** Representative images of the Transwell assay of HUVECs and MAECs treated as indicated. The Figure indicates the number of migrated cells. Scale bars = 100 μm. **D** Immunofluorescence analysis of CD31 (green) and α-SMA (red) double-stained HUVECs and MAECs, and the nuclei were stained with DAPI (blue). Scale bars = 100 μm. *p < 0.05, **p < 0.01, ***p < 0.001 and ****p < 0.0001 vs. control group; #p < 0.05, ##p < 0.01, ###p < 0.001 and ####p < 0.0001 vs. the TGF-β1 group

We next investigate whether SIRT1 was implicated in the inhibitory effect of DAPA on EndMT. We used two approaches to suppress SIRT1 expression in the presence of TGF-β1 combined with DAPA treatment, and the expression of proteins involved in EndMT was examined. DAPA-regulated amelioration of EndMT induced by TGF-β1 was dramatically inhibited by EX527 (Fig. 4A). Inhibition of SIRT1 significantly increased α-SMA and Vimentin expression, and markedly suppressed the expression of CD31 and VE-cadherin. In addition, the proliferation and migration of cells in each group were analyzed using EdU and Transwell assays. Endothelial cells were significantly activated and migrated upon TGF-β1 stimulation, and this effect was obviously inhibited by DAPA combined treatment. SIRT1 inhibition further enhanced endothelial cell proliferation and migration (Fig. 4B and C). The immunofluorescence results of CD31/ α-SMA also showed that DAPA-regulated amelioration of EndMT induced by TGF-β1 was dramatically inhibited by EX527 (Fig. 4D). Silencing of SIRT1 promoted cell proliferation and migration as well as exacerbated EndMT, which could counteract the effect of DAPA treatment (Fig. 4A–D).

### DAPA-mediated activation of SIRT1 improves heart function by reducing myocardial and perivascular fibrosis in mice induced by ISO

**Fig. 5:**
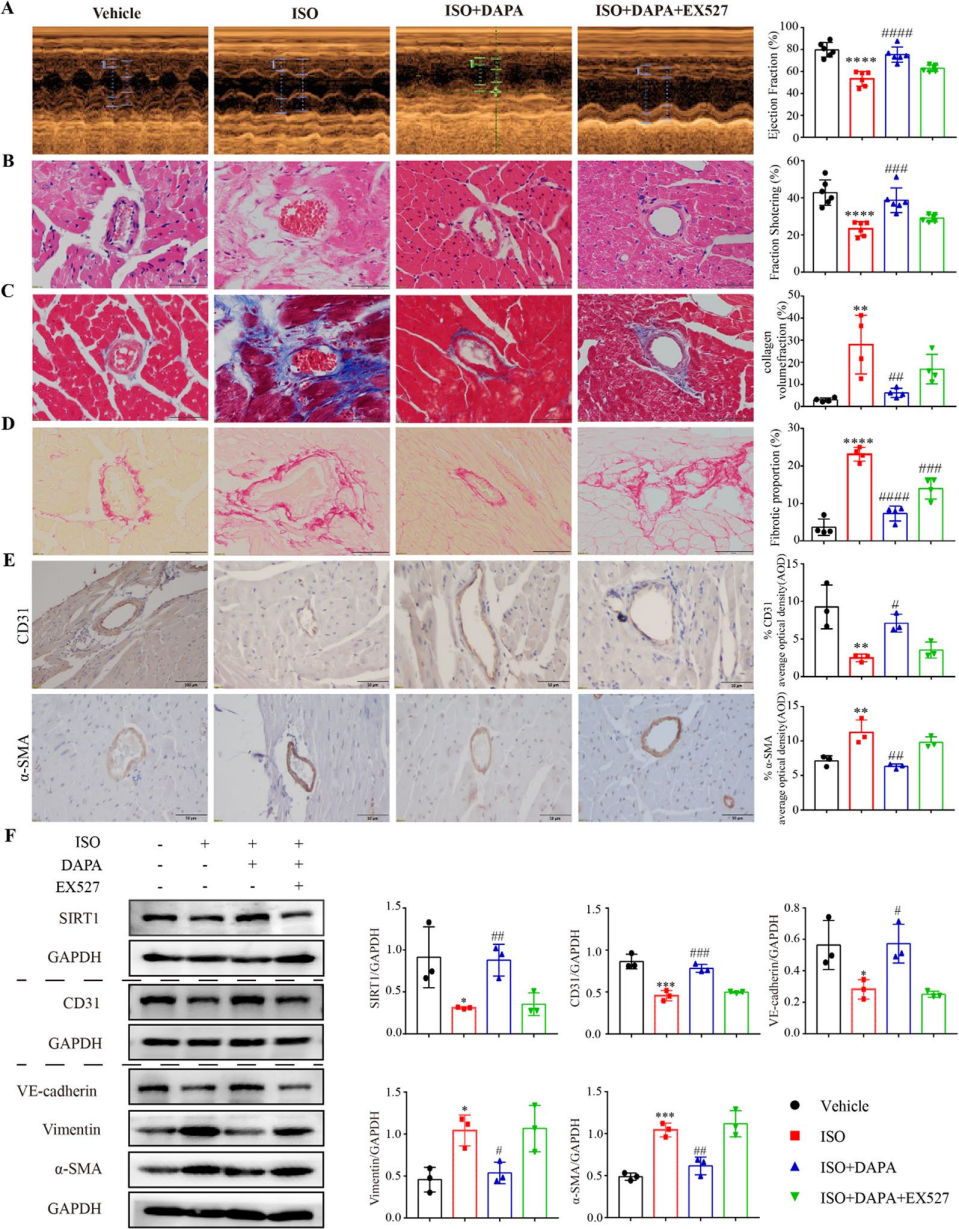
DAPA inhibits EndMT in ISO-induced mice by activating SIRT1. **A** Transthoracic echocardiogram in M-mode (n = 6). **B** H&E staining of the lengthwise section of the heart. Scale bar = 50 μm. **C** Representative images of Masson staining showing perivascular fibrosis in heart sections. Scale bar = 50 μm. **D** Representative images of PSR staining showing perivascular fibrosis in heart sections. Red represents collagen fiber deposition. Scale bars = 50 μm. **E** Representative IHC analysis for CD31 and α-SMA in the vascular wall in ventricular transverse sections from different groups of mice. Scale bars = 50 μm. **F** Western blotting analysis of EndMT-related proteins and SIRT1 in the mouse myocardium (n = 3) *p < 0.05, **p < 0.01, ***p < 0.001 and ****p < 0.0001 vs. Vehicle group; ^#^p < 0.05, ^##^p < 0.01, ^###^p < 0.001 and ^####^p < 0.0001 vs. the ISO group

We subsequently investigated the impact of DAPA-regulated SIRT1 on ISO-induced EndMT in mice. The SIRT1 inhibitor EX527 was administered in combination with ISO and DAPA. It was shown that EX527 was able to significantly suppress the cardioprotective effect of DAPA, in agreement with the results of in vitro experiments. EX527 treatment decreased the cardiac function of mice (Fig. 5A and B), and increased perivascular fibrosis (Fig. 5C and D) and EndMT as compared to the DAPA group (Fig. 5F). Immunostaining of CD31 and α-SMA of heart sections showed that ISO decreased the signal of CD31 and increased the signal of α-SMA (Fig. 5E). This abnormality was restored by treatment with DAPA, and the protective effect of DAPA was significantly diminished by application of EX527. These results suggest that DAPA can ameliorate EndMT and improve cardiac dysfunction in mice through SIRT1 activation.

### DAPA prevents the ubiquitination and degradation of SIRT1 by inhibiting TGF-β1-induced phosphorylation of SIRT1

**Fig. 6:**
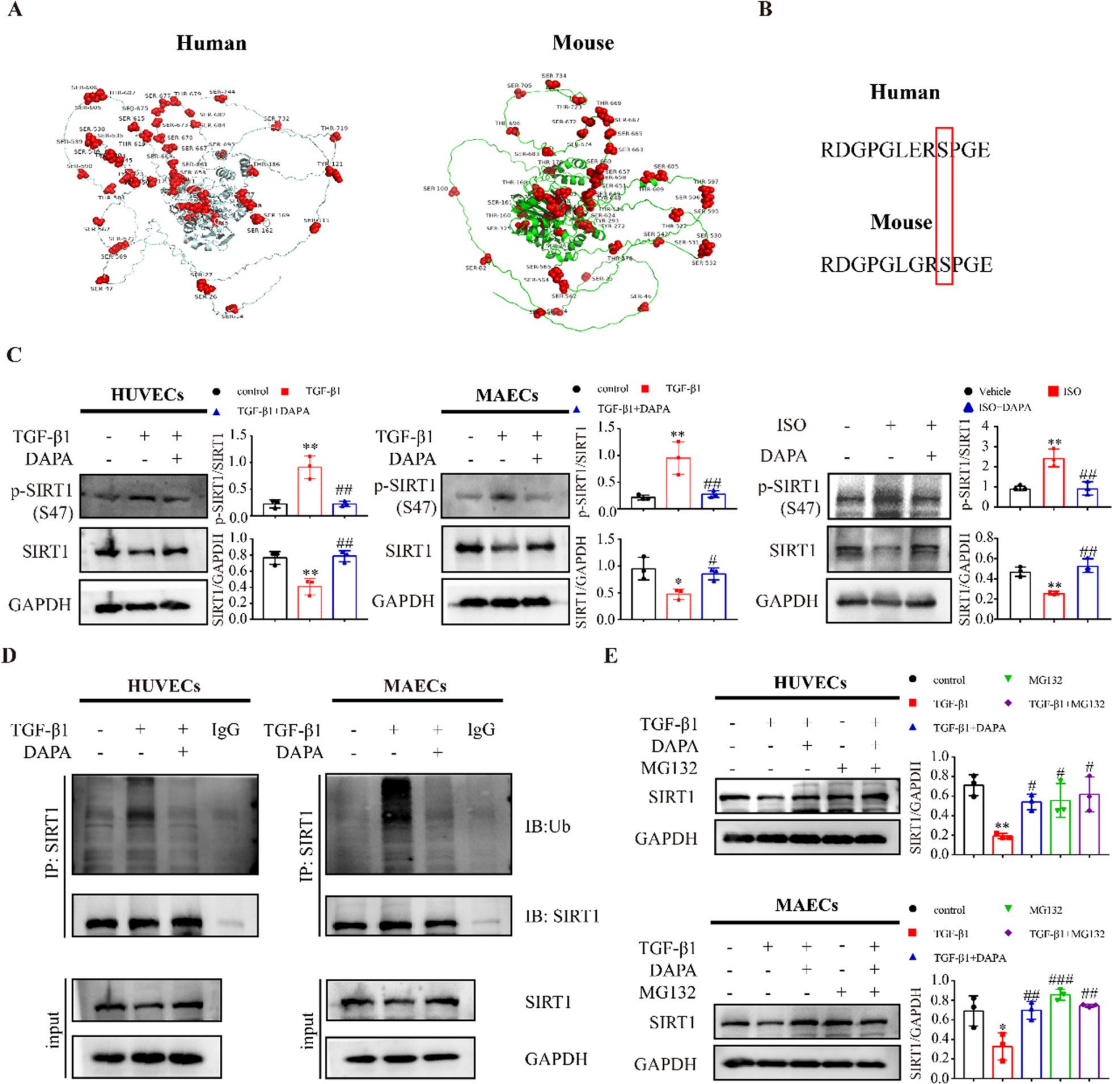
DAPA inhibits SIRT1 ubiquitination and degradation by inhibiting TGF-β1-induced SIRT1 phosphorylation. **A** The positions of phosphorylated residues on human and mouse SIRT1 were marked. **B** Shown is a sequence analysis of human and mouse SIRT1. The serine position is indicated by the number. **C** Representative Western blotting showing SIRT1 expression in vivo and in vitro treated as indicated and quantified. **D** SIRT1 immunoprecipitation reaction was performed in endothelial cells and subjected to Western blot analysis for detecting SIRT1 ubiquitination. **E** The effects of MG132 on SIRT1 protein expression upon TGF-β1. *p < 0.05, **p < 0.01 vs. control group; #p < 0.05 vs. the TGF-β1 group

It was reported that phosphorylation of SIRT1 is important for its function [24]. We first predicted and tagged the phosphorylation sites of human and mouse SIRT1 proteins via the NetPhos website (Fig. 6A). Since there is a substantial similarity of the amino acid sequences between human Ser-47 and mouse Ser-46 (Fig. 6B), SIRT1 phosphorylation levels at Ser-47 were examined in vivo and in vitro (Fig. 6C). It was shown that there was increased phosphorylation of SIRT1 at Ser47 and decreased expression of SIRT1 in endothelial cells upon TGF-β1 treatment, and these phenomena were prevented by DAPA combination treatment (Fig. 6C). In vivo experiment observed a similar phenomenon (Fig. 6C). Previous studies showed that phosphorylation-induced degradation of the protein could arise from the ubiquitination, which may account for the decreased expression of SIRT1 in the TGF-β1 group. Therefore, we performed Co-IP to investigate the ubiquitination level of SIRT1. We observed that SIRT1 was ubiquitinated under basal conditions and considerably increased after TGF-β1 treatment, which was attenuated by combined treatment with DAPA (Fig. 6D). In addition, we used the proteasome inhibitor MG132 to investigate whether increased ubiquitination gives rise to protein degradation induced by the proteasome, and the result showed that MG132 prevented the reduction of SIRT1 protein induced by TGF-β1 via inhibiting its degradation (Fig. 6E).

### DAPA promotes SIRT1 nucleus translocation through direct binding to SIRT1

**Fig. 7:**
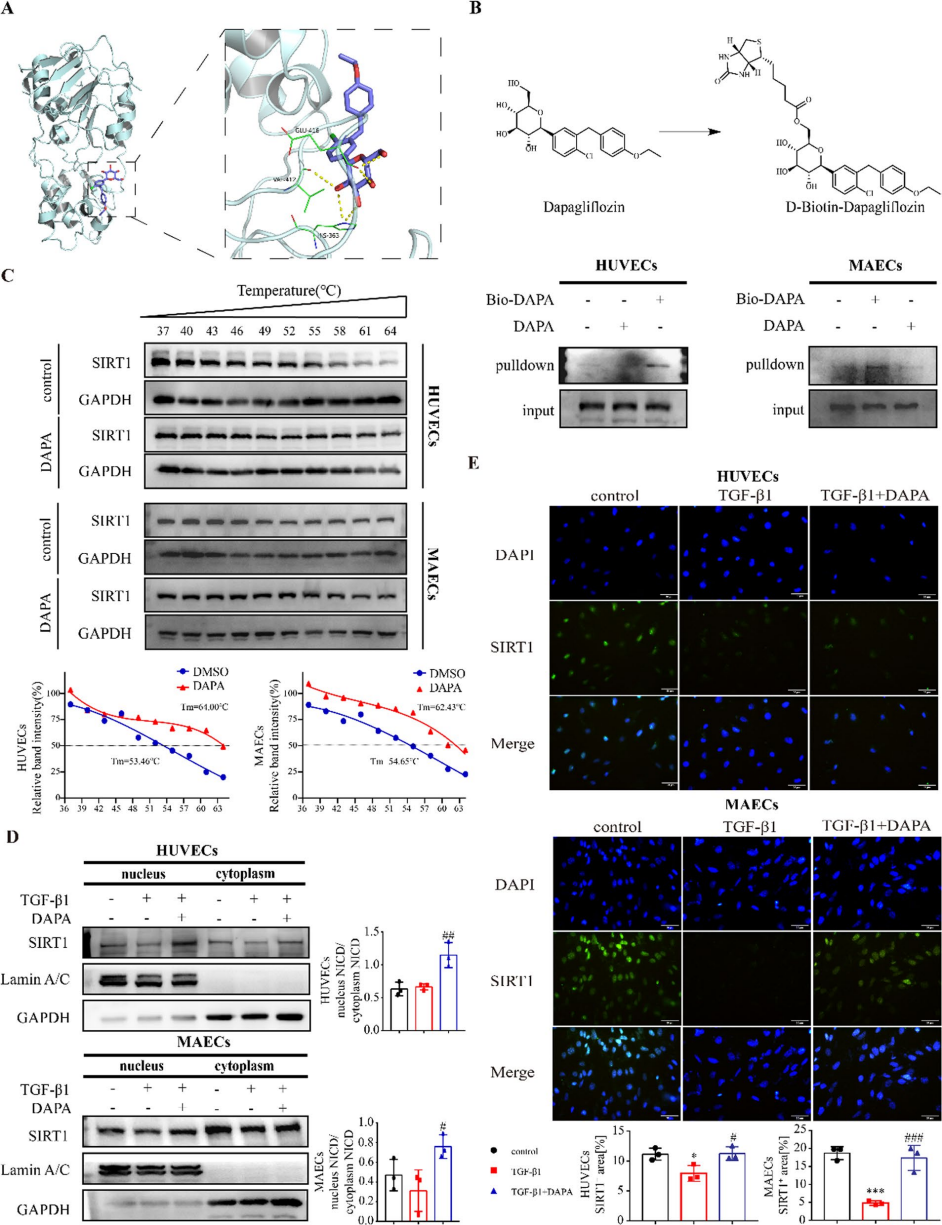
DAPA promotes nucleus translocation of SIRT1. **A** The overall binding view and local binding view of the SIRT1-DAPA complex obtained based on docking; the yellow dotted line represents the hydrogen bond, the green line represents the amino acid forming a hydrogen bond with the small molecule DAPA in the protein binding pocket, the cartoon represents the SIRT1 protein, and the purple stick is DAPA. **B** Chemical structure of DAPA and biotin-labeled DAPA (Bio-DAPA). The eluted product was obtained using a biotinylated protein interaction pull-down kit and further analyzed by Western blotting experiments. Total lysates were used as an input control. **C** CETSA between DAPA and SIRT1. The curve was fitted using GraphPad Prism 9.5.1. **D** Nucleus-cytoplasm distribution of SIRT1 in cells treated with TGF-β1and DAPA. **E** Immunofluorescent staining of SIRT1 in HUVECs and MAECs treated as indicated. Scale bars = 50 μm. *p < 0.05, **p < 0.01, and ***p < 0.001 vs. control group; #p < 0.05, and ##p < 0.01vs. the TGF-β1 group

To further clarify the mechanism of DAPA regulation in SIRT1 expression, we used AutoDock Vina 1.1.2 software to study DAPA and SIRT1 protein docking (Fig. 7A). In this simulation, the binding affinity of DAPA and SIRT1 protein was lower than − 5 kcal/mol, suggesting that DAPA and the SIRT1 protein have a high potential activity effect (Additional file 1: Figure S4A and B). Regarding the SIRT1-DAPA complex, the primary interaction between the SIRT1 protein and DAPA was hydrogen bonding and hydrophobic interactions, which might regulate the effect of DAPA on SIRT1 (Fig. 7A). We synthesized biotin-tagged DAPA and demonstrated through pull-down and western blotting experiments that SIRT1 is the binding protein of DAPA (Fig. 7B). CETSA found that DAPA directly binds to SIRT1 by affecting thermal stability (Fig. 7C).

It was reported that SIRT1 translocation from the cytoplasm to the nucleus is crucial for its activity [25]. Therefore, we measured SIRT1 expression in the nucleus and cytoplasm fractions and the results showed that treatment with TGF-β1 led to the translocation of SIRT1 from the nucleus to the cytoplasm, which was reversed by DAPA treatment in endothelial cells (Fig. 7D). Consistent results were observed by immunofluorescence staining of SIRT1 (Fig. 7E). These results suggest that DAPA increases SIRT1 nuclear translocation and the subsequent activation by directly targeting SIRT1.

### DAPA inhibits EndMT by regulating the Notch1 signaling pathway

**Fig. 8:**
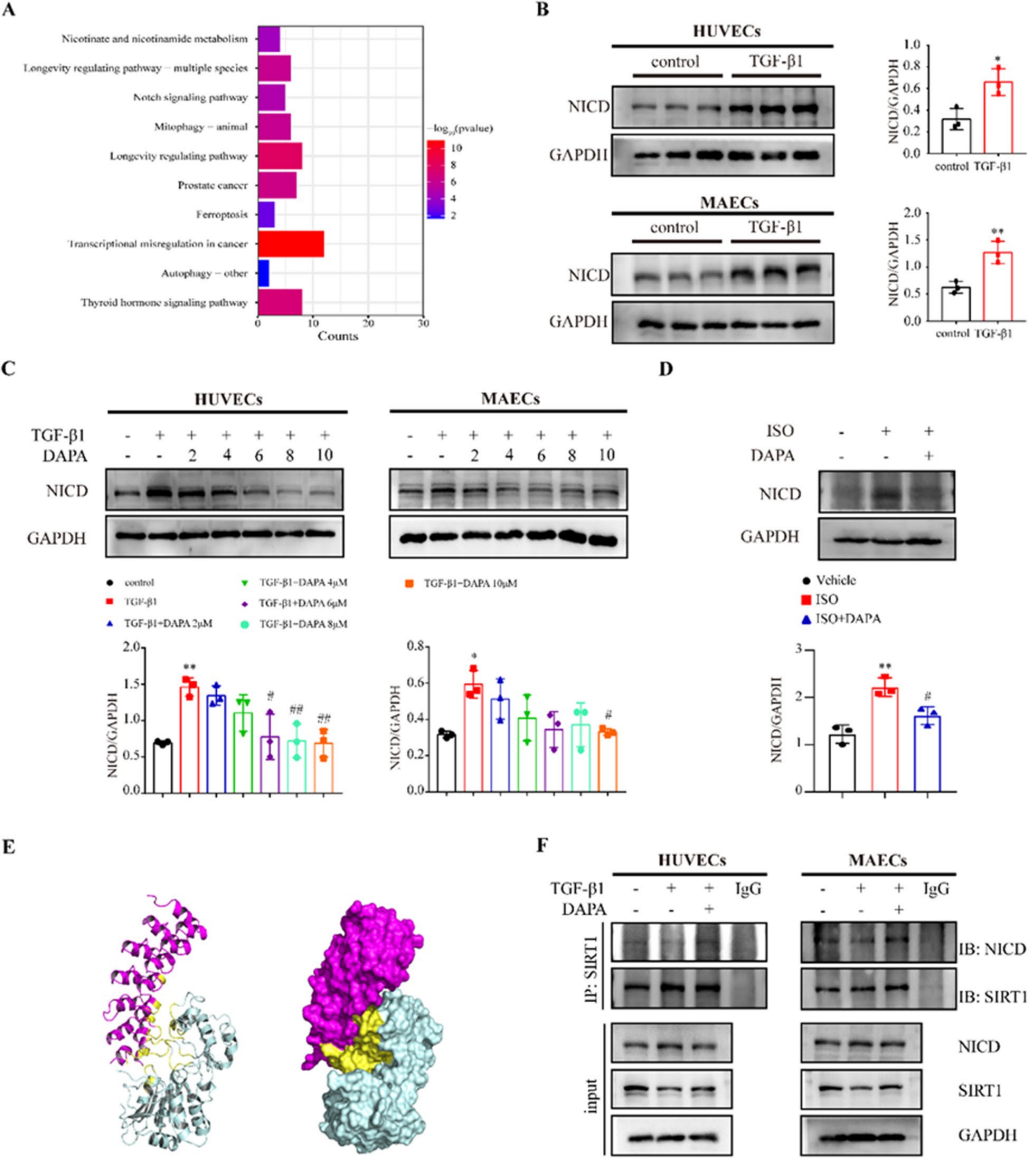
DAPA inhibits EndMT by regulating the Notch1 signaling pathway. **A** SIRT1 is implicated in the regulation of KEGG signaling pathways (Show only the top ten). **B** Representative Western blotting and quantitative analysis of NICD in HUVECs and MAECs treated with TGF-β1 for 48 h. **C** Representative Western blotting and quantitative analysis of NICD in HUVECs and MAECs treated with different concentrations of DAPA for 48h in the presence of TGF-β1. **D** Representative Western blotting showing NICD expression in vivo treated as indicated and quantified. **E** The binding conformation of SIRT1 and activated Notch1. The left image is the cartoon, the right image is the surface, and the yellow area is the binding region. **F** Co-IP of NICD and SIRT1. *p < 0.05, **p < 0.01, ***p < 0.001 and ****p < 0.0001 vs. control group; #p < 0.05, ##p < 0.01 vs. the TGF-β1 group

​To understand how SIRT1 regulates EndMT, we performed an enrichment analysis of the pathways in which SIRT1 is involved. It was demonstrated that SIRT1 might regulate Notch signaling pathway (Fig. 8A), which is a major signaling pathway involved in EndMT. Therefore, we focused on the regulatory relationship among SIRT1, Notch, and EndMT. The intracellular active segment of Notch1, NICD expression was increased, indicating that Notch1 was significantly activated in TGF-β1-induced EndMT (Fig. 8B). Combination treatment with DAPA decreased NICD expression in a dose-dependent manner, implying that DAPA may inhibit EndMT by regulating the Notch1 signaling pathway (Fig. 8C). We observed a significant increase in NICD in the ISO group in vivo, which was reversed after combined DAPA treatment (Fig. 8D). We then performed molecular docking with the SIRT1 protein as the receptor and activated Notch1 protein as the ligand (Fig. 8E). The binding affinity between SIRT1 and activated Notch1 was − 2.8 kcal/mol, suggesting a potential binding relationship between SIRT1 and activated Notch1 (Additional file 1: Figure S5A). The main binding between SIRT1 and activated Notch1 is a salt bridge, hydrogen bonds, and hydrophobic interactions (Additional file 1: Figure S5B). We validated the binding of SIRT1 and NICD proteins in endothelial cells using Co-IP, and the combined treatment with DAPA significantly increased the binding of SIRT1 to NICD (Fig. 8F). Thus, these results suggest that DAPA may inhibit EndMT by promoting the regulation of the Notch1 signaling pathway by SIRT1.

### DAPA enhances SIRT1 binding to NICD, contributing to NICD proteolysis through deacetylation

**Fig. 9:**
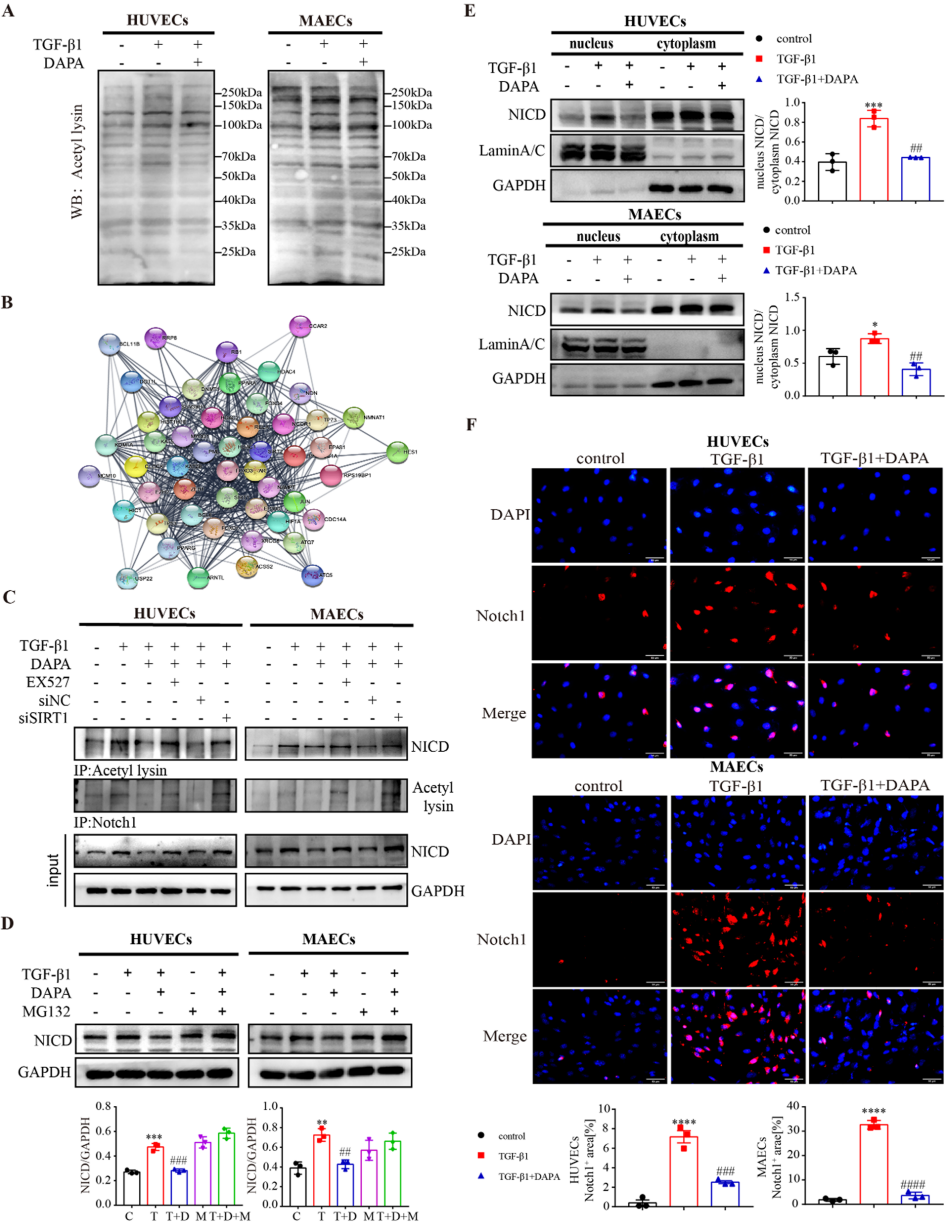
DAPA targets SIRT1 to deacetylate NICD and promote its proteolysis. **A** Immunoblot analysis of total acetylation level. **B** PPI networks of SIRT1. **C** NICD acetylation level was measured by IP using an anti-acetylation antibody and Western blotting. **D** The effects of MG132 on NICD protein expression upon DAPA. **E** Western blotting analysis of NICD expression levels in the nucleus and cytoplasm of HUVECs and MAECs treated as indicated and quantified. **F** Immunofluorescent staining of SIRT1 in HUVECs and MAECs treated as indicated. Scale bars = 50 μm. *p < 0.05, **p < 0.01, ***p < 0.001 and ****p < 0.0001 vs. control group; ^#^p < 0.05, ^##^p < 0.01, ^###^p < 0.001 and ^####^p < 0.0001 vs. the TGF-β1 group

There is compelling evidence indicating the crucial involvement of post-translational modification of NICD in the Notch1 signaling pathway [26, 27]. We assessed the acetylation levels in different cell groups using antibodies targeting pan-acetylated lysine. Our observations reveal a significant reduction in acetylation levels between 100 and 150 kDa following DAPA treatment, which coincides with the molecular weight range of NICD (Fig. 9A). Protein protein interaction network (PPI network) analysis demonstrated the participation of EP300, KAT family, HDAC family, and other proteins in SIRT1-regulated deacetylation and transduction of the Notch signaling pathway (Fig. 9B). These findings suggest that SIRT1 may suppress EndMT by directly binding to NICD and subsequently promoting its deacetylation. Subsequently, we investigated the acetylation level of NICD through Co-IP assay. TGF-β1 increased the acetylation level of NICD, and combined treatment with DAPA significantly decreased the acetylation (Fig. 9C). To further investigate the function of SIRT1 in DAPA-mediated regulation of NICD deacetylation, we inhibited SIRT1 expression levels using EX527 and siRNA, and found that inhibition of SIRT1 abolished the regulatory effect of DAPA on NICD (Fig. 9C).

Since NICD undergoes rapid ubiquitin-mediated degradation, which is inhibited by acetylation [28, 29]. The proteasome inhibitor MG132 was used to analyze the effect of SIRT1 on the degradation and stability of the NICD protein. MG132 treatment stabilized NICD protein and prevented the downregulation of NICD induced by DAPA treatment (Fig. 9D). In addition, previous studies have shown that NICDs induce transcription of downstream genes in the Notch  signaling pathway by transferring to the nucleus [30, 31]. Immunoblot analysis of isolated nucleus/cytoplasm fractions showed that NICD was mainly localized in the nucleus of the TGF-β1 group, whereas only a low proportion of NICD was localized in the nucleus of DAPA combined treatment group (Fig. 9E and F). These results suggest that NICD was deacetylated and degraded as a consequence of its interaction with SIRT1 in the nucleus.

## Discussion

Beyond their glucose-lowering effects, SGLT-2 inhibitors have been demonstrated to benefit the cardiovascular system in numerous clinical studies, making them a promising therapy for patients with HFrEF, with or without diabetes, and thus, have been recommended as the first-line treatment for HFrEF in several clinical guidelines [32, 33]. Clinical studies have demonstrated that DAPA exerts cardioprotective effects in patients with HFrEF irrespective of its glucose-lowering effects. This study aimed to investigate a novel mechanism through which DAPA improves cardiac function in non-diabetic mice with HF.

EndMT was significantly associated with myocardial interstitial and perivascular fibrosis, which was mitigated upon DAPA treatment, in a mouse model of ISO-induced cardiac dysfunction. Single-cell sequencing analysis of human healthy and HF tissues revealed that endothelial cell was significantly associated with the pathological progression of HFrEF. Furthermore, DAPA effectively restored the initial phenotype of endothelial cells by inhibiting EndMT. Based on these findings, DAPA was considered to attenuate myocardial and vascular fibrosis in non-diabetic mice by inhibiting EndMT. These findings revealed a potential mechanism underlying the beneficial effects of DAPA on HF in patients without diabetes.

EndMT, which occurs in endothelial cells exhibiting impaired cellular function, promotes the pathogenesis of vascular endothelial injury, vascular remodeling, and myocardial fibrosis. Early recovery of endothelial cell function is an effective therapeutic mechanism of anti-HF agents [34, 35]. Preclinical evidence and extensive clinical trial data suggest that DAPA alleviates endothelial dysfunction [9, 36–39]. In this study, DAPA significantly inhibited EndMT and alleviated cardiac dysfunction in ISO-treated mice, suggesting that DAPA is a potential therapeutic for patients with HFrEF exhibiting endothelial dysfunction or EndMT. Future studies must screen patients with HF exhibiting endothelial dysfunction or EndMT features to assess the clinical benefit of DAPA therapy.

SIRT1, an NAD^+^-dependent nicotinamide adenine deacetylase, exerts preventive effects on various cardiovascular diseases [12, 14, 15]. The findings of this study demonstrated that DAPA significantly restored the expression of SIRT1 in the ISO-induced cardiac dysfunction mouse model and the TGF-β1-induced EndMT model are consistent with those of previous studies [22, 40]. Molecular docking, pull-down assays, and CETSA experiments confirm the direct targeting effect of DAPA on SIRT1. In addition to DAPA, other types of SGLT-2 inhibitors, such as Ipragliflozin, Canagliflozin, and Empagliflozin, exhibit the up-regulation of SIRT1 expression and modulation of energy metabolism and mitochondrial function [41–43]. However, apart from our study, the direct targeting of SIRT1 by other SGLT-2 inhibitors has not been previously reported. Furthermore, this study proposed a potential strategy for future clinical investigations to augment SIRT1 activity and enhance responsiveness to DAPA treatment in patients with HFrEF through the co-administration of nicotinamide. Further studies are needed to examine the feasibility of this strategy.

Previous studies have demonstrated the DAPA upregulates the expression and activity of SIRT1. However, these studies focused on the protein level alterations and did not elucidate the regulatory mechanisms of SIRT1. This study demonstrated that DAPA inhibited the phosphorylation of SIRT1 at Ser47 and the ubiquitin-mediated degradation of SIRT1, resulting in the upregulation of SIRT1. Furthermore, previous studies have indicated that SIRT1 is located in both the cytoplasm and nucleus. The nucleus localization of SIRT1 is critical for its regulatory effects on downstream targets [44]. In particular, only nucleus SIRT1 is functional, promotes histone H3 deacetylation, and inhibits antimycin A-induced cell death and oxidative stress-induced cell death [45]. In this study, DAPA was demonstrated to upregulate SIRT1 expression, especially nucleus SIRT1 expression, using nucleus/cytoplasm protein fractionation and immunofluorescence assays. DAPA suppressed the ubiquitination and the subsequent degradation of SIRT1 by inhibiting SIRT1 phosphorylation at Ser47, as well as regulated SIRT1 expression and activity by promoting its nucleus translocation.

The activation of the Notch1 signaling pathway is essential for TGF-β1-induced EndMT. The upregulation of NICD acetylation mediates the activation of the Notch1 signaling pathway [26, 27, 46]. The Notch signaling pathway is reported to promote the progression of EndMT in human coronary endothelial cells and the development of atherosclerosis [47]. Our study revealed that DAPA inhibits EndMT through the SIRT1-regulated the Notch signaling pathway. Molecular docking analysis examined the ability of SIRT1 to bind activated Notch1 proteins and identified the potential binding sites. SIRT1, an intrinsic negative regulator of the Notch signaling pathway in endothelial cells, decreases NICD transcription and stability through deacetylation [26]. In this study, the combination treatment with DAPA suppressed the expression of NICD by enhancing the binding of SIRT1 to NICD and subsequently facilitating NICD acetylation and degradation in the EndMT model. Additionally, SIRT1 inhibition significantly mitigated the effects of DAPA. Notch, a type I transmembrane protein, undergoes three hydrolytic cleavages upon ligand binding, releasing NICD fragments into the cytoplasm. After nucleus translocation, NICD binds to target genes and regulates their transcription [30, 31]. Nucleus/cytoplasm fractionation and immunofluorescence analysis revealed that the DAPA-mediated regulation of NICD is specifically localized to the nucleus. The findings of this study demonstrated that the activated SIRT1-mediated regulation of NICD deacetylation is indispensable for the inhibitory effects of DAPA on EndMT and myocardial fibrosis in non-diabetic mice.

To more accurately simulate the effective dosage of DAPA in human subjects during animal studies, we consulted relevant study [17] and determined the final dosage for this study based on the body surface area-adjusted equivalent dose ratio (taking into account the recommended 10 mg/dose for HFrEF patients and DAPA's absolute oral bioavailability of 78%), which has been shown to significantly improve cardiac function in mice. However, oral absorption, blood concentration, and metabolism of DAPA vary between humans and mice. Hence, the optimal dosage to improve EndMT in mice must be investigated. In particular, the oral availability, plasma protein binding rate, and clearance half-life of DAPA must be identified in HF mice.

This study focused on the molecular mechanisms of cardiovascular effects of DAPA in HFrEF mice, but has not been able further to extend the study to other types of HF. Currently, heart failure with preserved ejection fraction (HFpEF) constitutes approximately 50% of the total cases of HF [48, 49], and its prevalence is increasing [50]. Several large trials have evaluated the efficacy of SGLT-2 inhibitors in patients with HFpEF. The DELIVER study and the EMPEROR Preserve trials both demonstrated significant efficacy of SGLT-2 inhibitors in patients with HFpEF [51, 52]. These trials represent a significant milestone and future research should focus on further elucidating the mechanism of action of SGLT-2 inhibitors in HFpEF patients. The subsequent phase of our research aims to provide animal findings that elucidate the underlying mechanisms through which DAPA ameliorates adverse outcomes in patients with HFpEF.

This study was for the first time to thoroughly explore the effects and specific mechanisms of the SGLT-2 inhibitors DAPA on EndMT in non-diabetic HF. Our results showed that DAPA inhibits EndMT and improves myocardial fibrosis in non-diabetic mice by activating SIRT1 and promoting its regulation of NICD deacetylation. Further research will contribute to elucidating the precise role of SGLT-2 inhibitors in cardiovascular disease protection, thereby establishing a comprehensive theoretical framework for identifying novel therapeutic targets in this domain, which is of great significance for promoting the understanding and development of the cardiovascular protective mechanism of SGLT-2 inhibitors in academia.

## Conclusion

The study revealed that DAPA can attenuate EndMT induced by ISO in non-diabetic HF mice. This beneficial effect is achieved through SIRT1-mediated deacetylation and degradation of NICD, which in turn inhibits the activation of the Notch1 signaling pathway. Furthermore, we found that DAPA treatment can ameliorate myocardial-perivascular fibrosis and cardiac dysfunction in these animals. These findings shed new light on the potential clinical applications of DAPA in the treatment of non-diabetic HF. By targeting the EndMT process and its associated mechanisms, DAPA may provide a promising therapeutic approach for HF. Moreover, based on the findings of this study, further research in this area will contribute to developing more effective treatments for patients suffering from non-diabetic HF.

### Supplementary Information

**Additional file 1.** **Fig. S1.** The increased signaling of Endothelial cell after HF. **Fig. S2.** The involvement of EndMT in the pathogenesis of heart failure was observed in TAC-induced mice. **Fig. S3.** The statistical results of Western blot analysis (A) and EdU incorporation assay (B) are presented in Figure 3.**Fig. S4.** Molecular docking data of DAPA and SIRT1.**Fig. S5.** SIRT1 docking data with activated Notch1 molecules.**Fig. S6.** Immunofluorescent staining of SGLT2 in HUVECs and MAECs treated as indicated (in response to reviewer comments).

## Data Availability

All data generated or analyzed during this study are included in this published article and its Additional files.

## Abbreviations

EndMT: Endothelial–mesenchymal transition
SGLT-2: Sodium–glucose-linked transporter 2
DAPA: Dapagliflozin
HF: Heart failure
NICD: The active segment of Notch1
HFrEF: Heart failure with reduced ejection fraction
ISO: Isoproterenol
HUVECs: Human umbilical vein endothelial cells
MAECs: Mouse aortic endothelial cells
Co-IP: Co-immunoprecipitation
IHC: Immunohistochemistry
PSR: Picric acid-Sirius red
LVFS: Left ventricular fractional shortening
LVEF: Left ventricular ejection fraction
siRNA: Small interfering RNA
CETSA: Cellular thermal shift assay
HFpEF: Heart failure with preserved ejection fraction
