# Supplementary material for: SIRT1 mediates the inhibitory effect of Dapagliflozin on EndMT by inhibiting the acetylation of endothelium Notch1
Source: Cardiovasc Diabetol. 2023 Nov 28;22:331. doi: 10.1186/s12933-023-02040-x (PMC10685714; doi:10.1186/s12933-023-02040-x)
Supplement: Supplementary file 1 — Additional file 1. Fig. S1. The increased signaling of Endothelial cell after HF. Fig. S2. The involvement of EndMT in the pathogenesis of heart failure was observed in TAC-induced mice. Fig. S3. The statistical results of Western blot analysis (A) and EdU incorporation assay (B) are presented in Figure 3.Fig. S4. Molecular docking data of DAPA and SIRT1.Fig. S5. SIRT1 docking data with activated Notch1 molecules.Fig. S6. Immunofluorescent staining of SGLT2 in HUVECs and MAECs treated as indicated (in response to reviewer comments). [file 12933_2023_2040_MOESM1_ESM.docx]

**Supplementary Information**

**SIRT1 mediates the inhibitory effect of Dapagliflozin on EndMT by inhibiting the acetylation of endothelium Notch1**

Weijie Wang, MD ^a, b,1^ Yilan Li, MD, Ph.D. ^a, b,1^ Yanxiu Zhang, MD ^a, b^ Tao Ye^e^ Kui Wang^e^ Shuijie Li, Ph.D. ^c, d, *^ Yao Zhang, MD, Ph.D. ^a, b, *^

**Author Affiliations:**

^a^ Department of Cardiology, The Second Affiliated Hospital of Harbin Medical University, Harbin, China

^b^ Key Laboratory of Myocardial Ischemia, Ministry of Education, Harbin Medical University, Harbin, China

^c^ Department of Biopharmaceutical Sciences, College of Pharmacy, Harbin Medical University, Harbin, China

^d^ State Key Laboratory of Frigid Zone Cardiovascular Diseases （SKLFZCD）, Harbin, China

^e^ Department of Organic Chemistry, College of Pharmacy, Harbin Medical University, Harbin, China.

**^1^ Weijie Wang and Yilan Li are equal first authors**

*** Corresponding authors:**

Correspondence to Dr. Yao Zhang (lead contact), Department of Cardiology, The Second Affiliated Hospital of Harbin Medical University, Key Laboratory of Myocardial Ischemia, Chinese Ministry of Education, 246 Xuefu Road, Harbin 150086, China. E-mail: [yaozhang_grace@163.com](mailto:yaozhang_grace@163.com)

or Shuijie Li, Department of Biopharmaceutical Sciences, College of Pharmacy, Harbin Medical University, Harbin, 150081, China. E-mail: [shuijie.li@hrbmu.edu.cn](mailto:shuijie.li@hrbmu.edu.cn)

**The dose and route of administration in animal experiments**

In the ISO group, ISO (Sigma, USA) was injected subcutaneously at 10 mg/kg/day for 3 days and then at 5 mg/kg/day for 11 days. In the other two groups, DAPA (MCE, USA) (1 mg/kg/day) was administered by gavage for 28 days, while EX527 (MCE, USA) (5 mg/kg/day) was administered by gavage in addition to ISO plus DAPA for 14 days in the combined EX527 group. After treatment, the mice were weighed and anesthetized, echocardiography was performed to determine cardiac structure and function, and blood and heart samples were collected.

**Detailed analysis methods for single-cell sequencing data**

*1.1 Combined data set*

First, merge the Seurat objects with the merge function, then use “Find Variable Features” of the Seurat R package (v4.1.0) to select 2000 highly variable genes, and then use “SCTransform” of the Seurat R package (v4.1.0). “Find Integration Anchors” and “Integrate Data” functions integrate a single cell data set. In addition, the “Run Harmony” function of the harmony R package (v0.1.1) is utilized to remove batch effects. The processed data were analyzed as principal components using “Run PCA” and then visualized using UMAP or TSNE.

*1.2 Cell clustering and annotation*

Using “Find Neighbors” and “Find Clusters” of Seurat R package (v4.1.0) (resolution set to 0.4, 0.6, 0.8, 1,1.2), cell clustering analysis of the above data set was performed (16 cell clusters for human, respectively). Finally, the resolution of 0.4 was selected as the parameter to divide the cell population. Cell type annotation refers to the marker genes given in the published articles on the above data set. Finally, the human data sets had 6 major cell groups, respectively.

*1.3 Cell population proportion statistics*

The single-cell data of the above-annotated cell groups were analyzed. The ggplot2 R package (v3.4.0) was used to display the results in pie charts, pile charts, and bar charts.

*1.5 Differential gene screening and functional analysis*

The “Find Markers” function of Seurat R (v4.1.0) (ident.1 = " HF ", ident.2 = "Normal heart") was used to analyze the differences between different cell groups. Differential genes were screened by setting filtering parameters (p-value < 0.05, absolute value of log2 (fold-change) > 1.2, occurrence percentage of gene_percentage_cell gene in the group >0.3). Finally, the ggplot2 R package (v3.4.0) was used to display the results in a rose chart. The obtained human differential genes were placed on Metascape (v3.5) (http://metascape.org) for gene enrichment analysis (selection of gene sets suitable for human). Finally, the enrichment GO term results of the top 10 (sorted according to the enrichment score) were selected and the results were displayed by bubble map.

*1.4 Data Processing*

The R-based GEO online tool GEO2R (http://www.ncbi.nlm.nih.gov/geo/geo2r/) was used to identify the differentially expressed genes (DEGs) between HF and sham-treated mice hearts tissue expression profiles, with the thresholds of |log2 (fold-change) | > 1.0 and p-value < 0.05.

*1.5 Cell-cell communication analysis*

CellChat R package (v1.6.1) was used to calculate the results of intercellular communication for human HF and Normal heart respectively (steps calculated respectively: 1. Build cellchat objects; 2. Select cellchatDB with Secreted Signaling receptor; 3. Data preprocessing, such as identifying overexpressed genes; 4. Calculate the communication probability between cell groups; 5. Construct a cell group communication network diagram).

**The increased signaling of Endothelial cell after HF**

**Fig. S1 The increased signaling of Endothelial cell after HF.**

**The involvement of EndMT in the pathogenesis of heart failure was observed in TAC-induced mice**

**Fig. S2**. The involvement of EndMT in the pathogenesis of heart failure was observed in TAC-induced mice. (A). Results for the GSEA enrichment of DEGs. (B). Enrichment analysis of biological processes involved in regulating EMT or EndMT-related pathways. (C) Enrichment analysis of KEGG signaling pathways involved in regulating EMT or EndMT-related pathways. (D) Enrichment of EMT or EndMT marker genes.

**The statistical results of Western blot analysis (A) and EdU incorporation assay (B) are presented in Figure 3**

**Fig. S3** The statistical results of Western blot analysis (A) and EdU incorporation assay (B) are presented in Figure 3. (A) Quantitative analysis of proteins. (B) Quantitative analysis of EdU.

**Molecular docking data of DAPA and SIRT1**

**A**

| Protein | coordinate of the box center (X, Y, Z) | the side length of the box  (X, Y, Z) |
| --- | --- | --- |
| SIRT1 | -24.630，51.710，-13.772 | 62.0，68.0，68.0 |

**B**

| Target name | Ligand name | Docking score(kcal/mol) |
| --- | --- | --- |
| SIRT1 | Dapagliflozin | -8.1 |

**Fig. S4** Molecular docking data of DAPA and SIRT1. (A) The protein's center coordinates and docking box parameters. (B) Based on AutoDock Vina docking of small molecules, the protein binding affinity was assessed (kcal/mol).

**SIRT1 docking data with activated Notch1 molecules**

**A**

| Receptor protein | Ligand protein | | Binding affinity (kcal/mol) |
| --- | --- | --- | --- |
| SIRT1 | | Notch1 | -2.8 |

**B**

**Fig. S5** SIRT1 docking data with activated Notch1 molecules. (A) Based on AutoDock Vina docking of small molecules, the protein binding affinity was assessed (kcal/mol). (B) SIRT1 protein and Notch1 interaction diagram.

**Immunofluorescence results of SGLT2 in endothelial cells**

**Fig. S6** Immunofluorescent staining of SGLT2 in HUVECs and MAECs treated as indicated (in response to reviewer comments). Scale bars = 50 μm. *p < 0.05, **p < 0.01vs. control group; #p < 0.05, and ##p < 0.01vs. the TGF-β1 group.
